# Tolvaptan for hyponatremia with preserved sodium pool in critically ill patients

**DOI:** 10.1186/s13613-015-0096-2

**Published:** 2016-01-04

**Authors:** Michele Umbrello, Elena S. Mantovani, Paolo Formenti, Claudia Casiraghi, Davide Ottolina, Martina Taverna, Angelo Pezzi, Giovanni Mistraletti, Gaetano Iapichino

**Affiliations:** Unità Operativa di Anestesia e Rianimazione, Azienda Ospedaliera San Paolo-Polo Universitario, Via A. Di Rudinì, 8, 20142 Milan, Italy; Dipartimento di Fisiopatologia Medico-Chirurgica e dei Trapianti, Università degli Studi di Milano, Milan, Italy

**Keywords:** Hyponatremia, Critical illness, Tolvaptan

## Abstract

**Background:**

Hyponatremia is the most common electrolyte disturbance in hospitalized patients, and it represents a well-established risk factor for ICU/hospital mortality. The majority of hyponatremic states are associated with elevated arginine vasopressin levels and a preserved sodium pool. Conventional treatment is either not pathophysiologically oriented or of limited effectiveness. The aim of the present study is to investigate the use of enteral Tolvaptan in critically ill hyponatremic patients.

**Methods:**

This is a retrospective observational study in a general ICU. Patients with preserved sodium pool hyponatremia refractory to conventional therapy were enrolled. The hemodynamic, renal, and hepatic functions, together with sodium and water balance as close as possible to the drug administration and up to 72 h thereafter, were analyzed. The main outcome was a serum sodium increase of ≥ 4 mmol/L in 24 h; secondary endpoints were the ability to maintain serum sodium at 24 and 72 h, a decrease in urine sodium concentration and an increase in sodium-free diuresis.

**Results:**

38 patients were enrolled. The average dose of enteral Tolvaptan was 7.5 mg. 31 patients (81.6 %) increased their serum sodium >4 mmol/l/24 h; the average increase was 6.7 ± 3.4 mmol/l during the first 24 h (*p* < 0.001 vs baseline), and this was sustained at 72 h. No adverse effects were reported. Plasma sodium (*R* = −0.622, *p* < 0.001), urine sodium (*R* = −0.345, *p* < 0.001), central venous oxygen saturation (*R* = 0.401, *p* = 0.013), and BUN (*R* = −0.416, *p* = 0.031) before Tolvaptan were all significantly correlated with the absolute increase in serum sodium after the administration.

**Conclusions:**

Enteral administration of Tolvaptan seems effective in the treatment of hyponatremia with preserved sodium pool in critically ill patients. Even if the study was underpowered to detect significant side effects or complications of unwarranted fast corrections of hyponatremia, we report no complications.

**Electronic supplementary material:**

The online version of this article (doi:10.1186/s13613-015-0096-2) contains supplementary material, which is available to authorized users.

## Background

In a large-scale epidemiologic study, hyponatremia (serum sodium concentration–[Na^+^] ≤ 135 mmol/L) was present at admission in 14.5 % of patients, and during hospitalization in an additional 5 % [1]. The risk of mortality in those patients is evident even in mild cases ([Na^+^] 130–134 mEq/L), which constitute the majority of cases of hyponatremia, and the increased risk of death persists up to 5 years beyond discharge [1].

In the ICU, the prevalence of hyponatremia on admission is around 15–20 % [2, 3]; moreover, among patients with normal [Na^+^] at admission, a first episode of ICU-acquired hyponatraemia develops in 11 % of cases [4]. Independent of the severity of the underlying disease, both hyponatremia at admission and ICU-acquired hyponatremia are significantly related to ICU and hospital mortality. Similar associations with mortality result from the duration of sodium disturbances [3, 4]. Correction of acute and severe hyponatremia is lifesaving; however, despite the widespread clinical impression that correction of less severe hyponatremia is also worthwhile, evidence-based data demonstrating clinical benefit are limited [5].

[Na^+^] represents the ratio of total body sodium pool to total body water pool [6]. Depending on total body sodium stores, hyponatremia can be hypovolemic, normovolemic, or hypervolemic, with body sodium pool reduced, preserved, or increased, respectively. However, the majority of hyponatremic states in critical illness are frequently non-hypovolemic (e.g., syndrome of inappropriate antidiuresis–SIAD) [2] and likely associated to elevated plasma levels of arginine vasopressin (AVP) [7], due to an underlying cause of inappropriate antidiuresis [8]. AVP-induced water retention leads to increased body water pool and subsequent dilution of serum sodium [9, 10], with excess total body water relative to the preserved total body sodium pool [11].

The traditional approach to the treatment of such cases involves restriction of free water intake [12], use of loop diuretics [13], demeclocycline [14], osmotic diuretics, such as urea [15] or intravenous administration of normal or hypertonic saline [16]. In ICU patients, fluid restriction is in fact poorly tolerated and difficult to achieve [17], and increased intake of salt does not address the causative factor, which is free water excess.

Indeed, loop diuretics result in the excretion of urine with a lower sodium concentration than plasma (i.e., more water than sodium is excreted, as compared to plasma) [13]. The subsequent correction of hyponatremia would then occur at the expense of salt depletion. For this reason, it is generally recommended that a concomitant administration of both NaCl (as hypertonic saline) and furosemide is performed in hyponatremia with normal sodium pool [18]. In particular, this is the only effective approach in severe cases of hyponatremia (i.e., coma and or convulsions), because the effects of combined salt and furosemide are extremely rapid [11]. However, other complications besides the reduction in body sodium pool can be determined by loop diuretic administration, such as hypokalemia, hypochloremia, hypomagnesemia, metabolic alkalosis [19], and this explains why this treatment is generally reserved to the acute phases of correction only [20].

Urea is an osmotic diuretic, and it increases free water excretion according to each patient urine osmolality and the amount of drug administered. Despite recent positive report on its use in critically ill patients [21], clinical data with this drug are, however, limited; in addition, urea is not a receptor-targeted agent and it has been associated with potential side effects (azotemia, nausea/vomiting, allergic reactions, renal toxicity, hypernatremia, and/or hypokalemia) [21].

Vaptans are a recently introduced class of vasopressin V2-receptor antagonists, yielding to an increased electrolyte-free water excretion and, thus, increased serum sodium concentration [22]. Several studies showed their safety and effectiveness in hyponatremia with preserved total body sodium pool in different categories of patients [17, 23–27]; however, the experience with these drugs is limited in the critical care setting. After positive initial experience with oral Tolvaptan in single cases [28, 29], we sought to analyze our case series to investigate the short-term outcomes on water and electrolyte balance for patients prescribed oral vaptans for hyponatremia with preserved total body sodium pool within our ICU.

## Methods

### Ethics, consent, and permission

The case series was conducted in a in a 6-bed general ICU of a University hospital. After obtaining approval from the Institutional Review Board (“Comitato Etico Interaziendale Milano Area A”—n. 16413), we enrolled patients treated with oral Tolvaptan between the date the drug was introduced into our clinical practice, January 2012, and January 2014. In all cases, decision to administer Tolvaptan was made by the physician in charge of the patient. Patients provided informed consent for the utilization of their data. All patient data have been anonymized.

### Data collection and inclusion criteria

We performed a retrospective analysis of prospectively collected case series; patients were treated following a specific internal protocol for the management of hyponatremia, resulting in a heterogeneous population of critically ill patients in whom hyponatremia was treated homogeneously. The following inclusion criteria were used: (1) diagnosis of clinically significant hypoosmolar hyponatraemia ([Na^+^] ≤ 135 mmol/L and serum osmolality <280 mOsm/kg) with raised urine osmolality (urine osmolality >100 mOsm/kg and urine sodium >20 mmol/L) [30]; (2) no history of reduction in body sodium pool (i.e., diuretic use, gastrointestinal losses, hemorrhage, low sodium intake); (3) lack of clear signs of hypovolemia (i.e., tachycardia, pale, cold, and clammy skin, delayed capillary refill, hypotension, oliguria, hyperlactatemia, reduced central venous blood oxygen saturation); (4) the presence of a possible underlying cause of inappropriate antidiuresis (i.e., central nervous system disorder, pulmonary diseases, mechanical ventilation, drug-induced) [8]; and (5) failure to correct hyponatraemia (absolute increase in [Na^+^] ≥ 4 mmol/L over baseline) despite at least 24 h of free water restriction (<1 l); (6) age >18.

Given the limited experience with the drug in this class of patients and to avoid the risks of overcorrection, fluid restriction was only prescribed before the administration of Tolvaptan. As in case of serum sodium overcorrection, any saline infusion was temporarily withheld, and further correction from urinary free water losses was prevented by replacing losses with 5 % dextrose in water [11]. The dose of Tolvaptan was not standardized as the decision on the dose to be administered was left to the attending intensivist. We performed a post hoc subgroup dividing patients by the dose they receive (either 7.5 or 15 mg).

Patients who were given Tolvaptan were identified from pharmacy records; we then retrieved their paper charts and analyzed their clinical course. The data collected included age, sex, diagnosis, ICU and hospital length of stay, dose and duration of Tolvaptan administration, Sequential Organ Failure Assessment (SOFA) score, ICU, and in-hospital mortality. Hemodynamic data including mean arterial pressure, heart rate, arterial lactate, base excess, rates of inotrope, and vasopressor infusions were recorded, as was 24-h urine output and the net fluid balance, the total amount of sodium administered and the daily sodium balance, the duration of hyponatremia prior to treatment and the day of Tolvaptan administration from ICU admission, serum and urine sodium and potassium at initiation of treatment and every 24 h. Additional laboratory data included serum creatinine, blood urea nitrogen (BUN), liver function tests, albumin, and hemoglobin concentration. Osmolality in serum and urine was not formally measured, and it was calculated using a standard formula; only 17 patients had urinary nitrogen determinations. We thus decided to present two different pieces of information on urinary osmolality: on the one hand, we calculated urinary salt osmolality (i.e., twice the sum of urinary sodium and potassium), which was available in all patients; on the other hand, for the subgroup of patients in which this was available, we also calculated, with a standard formula, total urinary osmolality. Data were collected as close as possible to the drug administration, and up to 72 h thereafter where this was available. Adverse events including hypotension and excessive rise in sodium concentration were recorded.

### Outcomes

The primary outcome was the efficacy of Tolvaptan in correcting hyponatremia, defined as the percentage of patients with an absolute increase in [Na^+^] ≥ 4 mmol/L over baseline at 24 h after administration of the drug. This was selected for consistency with previous reports of similar interventions [31–33]. Secondary outcomes included absolute increase in [Na^+^] at 24 h, absolute increase of [Na^+^] at 72 h, and absolute decrease in urine sodium concentration. Sodium-free water clearance was estimated from an established equation using urine volume and urine and serum sodium [34]. As a safety endpoint, the rate correction of hyponatremia was examined. A rapid correction was defined as any increase in [Na^+^] ≥ 12 mmol/L after 24 h [11]. We also searched for adverse reactions resulting from hypovolemia and increased diuresis such as hemodynamic compromise and effects on serum potassium and renal function. An exploratory analysis was also conducted, to assess possible pre-treatment variables that could predict the response to therapy.

### Statistical analysis

Data are represented as mean values ± standard deviation if variables are normally distributed (as assessed by Shapiro–Francia test), or median (interquartile range) if not. Due to the observational nature of the study in a field for which no data are available as for the expected effect size, we referred to a study in which a vaptan was administered to a population of non-critically ill patients with hyponatremia with preserved total body sodium pool [31] to estimate the power. In that study, the percentage of patients administered a vaptan vs. placebo who increased their [Na^+^] ≥ 4 mmol/l in 24 h was 58 vs. 11 %. Considering a similar effect size and since we enrolled 38 patients, our study yields a power of 80.4 % with *α* = 0.05.

Paired Student’s *t* test, or the Wilcoxon signed-rank test, was used for the comparison of variables between baseline and 24 h after Tolvaptan administration. The time course of serum sodium over the first 72 h was studied with Friedman test with post hoc comparison test. The association between the absolute increase in [Na^+^] over the first 24 h and possible predictors of the response was assessed by means of parametric or non-parametric correlation analysis.

Data were collected from the medical charts and stored in an electronic data abstraction form, using a Microsoft Excel 2010 spreadsheet (Microsoft Corporation, Redmond, Wash). Statistical analysis was performed with Stata/SE 12.0 (StataCorp, College Station, TX USA) statistical software. For all comparisons, *p* < 0.05 was considered significant.

## Results

### Demographics and outcome

38 patients received enteral Tolvaptan. Demographics and clinical characteristics at ICU admission and at study enrollment, as well as ICU and hospital outcomes, are reported in Table 1. Average [Na^+^] at ICU admission was 135 [131; 138] (range 113–148). Hyponatremia was diagnosed on average on day 5 [2, 12] from ICU admission; nadir [Na^+^] was 132 [131; 134]. Tolvaptan was administered on day 8 [4, 13].

**Table 1:** Patient demographics and clinical data at enrollment

Age (years)	53 ± 15
Male sex	24 (63.1 %)
Height (cm)	170 ± 9
Body weight (kg)	76 ± 21
Ideal body weight (kg)	63 ± 7
Body mass index (kg/m2)	26 ± 6
Simplified acute physiology score II	26 ± 12
Admission type	
Medical	26 (68.4 %)
Surgical unscheduled	12 (31.6 %)
Diagnosis	
Pneumonia	12 (31.6 %)
Urosepsis	6 (15.8 %)
Peritonitis	5 (13.2 %)
TUR syndrome	4 (10.5 %)
Acute pancreatitis	3 (7.9 %)
Cardiogenic shock	2 (5.3 %)
Drug abuse	2 (5.3 %)
Meningitis	2 (5.3 %)
Osteomyelitis	1 (2.6 %)
Chest trauma	1 (2.6 %)
SOFA score at enrollment	3 [1; 5]
Organ support at enrollment	
Mechanical ventilation	38 (100 %)
Vasoactive therapy	2 (5.3 %)
Worst SOFA score during ICU stay	6 (3; 8)
Length of ICU stay (days)	14 (6; 23)
ICU mortality	7 (18 %)
Hospital mortality	12 (31 %)
Age (years)	53 ± 15
Male (sex)	24 (63.1 %)
Height (cm)	170 ± 9

### Tolvaptan dosing, efficacy, and adverse effects

The average dose of Tolvaptan administered was 7.5 [7.5; 15] mg enterally: 24 patients (63 %) received 7.5 mg, while the remaining 14 (37 %) received 15 mg. Additional file 1: Tables S1 and S2 summarize the results of the subgroup analysis according to the dose of Tolvaptan received. We did not find any relevant difference between patients receiving 7.5 vs. 15 mg.

Thirty-one (81.6 %) patients met the criterion for successful response, defined as an absolute serum sodium increase ≥4 mmol/L 24 h after Tolvaptan administration. Table 2 shows the primary and secondary outcomes of the study.

**Table 2:** Primary and secondary outcomes

Primary outcome	
≥4 mmol/l increase in serum sodium over baseline at 24 h—n (%)	31/38 (81.6 %)
Secondary outcomes	
Absolute increase in serum sodium over baseline at 24 h—mmol/l	6.7 ± 3.4
Absolute increase in serum sodium over baseline at 72 h—mmol/l	5.5 ± 3.7
Absolute reduction in urine sodium over baseline at 24 h—mmol/l	-68.0 ± 39.9
≥12 mmol/l increase in serum sodium over baseline at 24 h—n (%)	4/38 (10.5 %)
Average hourly increase in serum sodium—mmol/l*h	0.28 ± 0.14

**Fig. 1:**
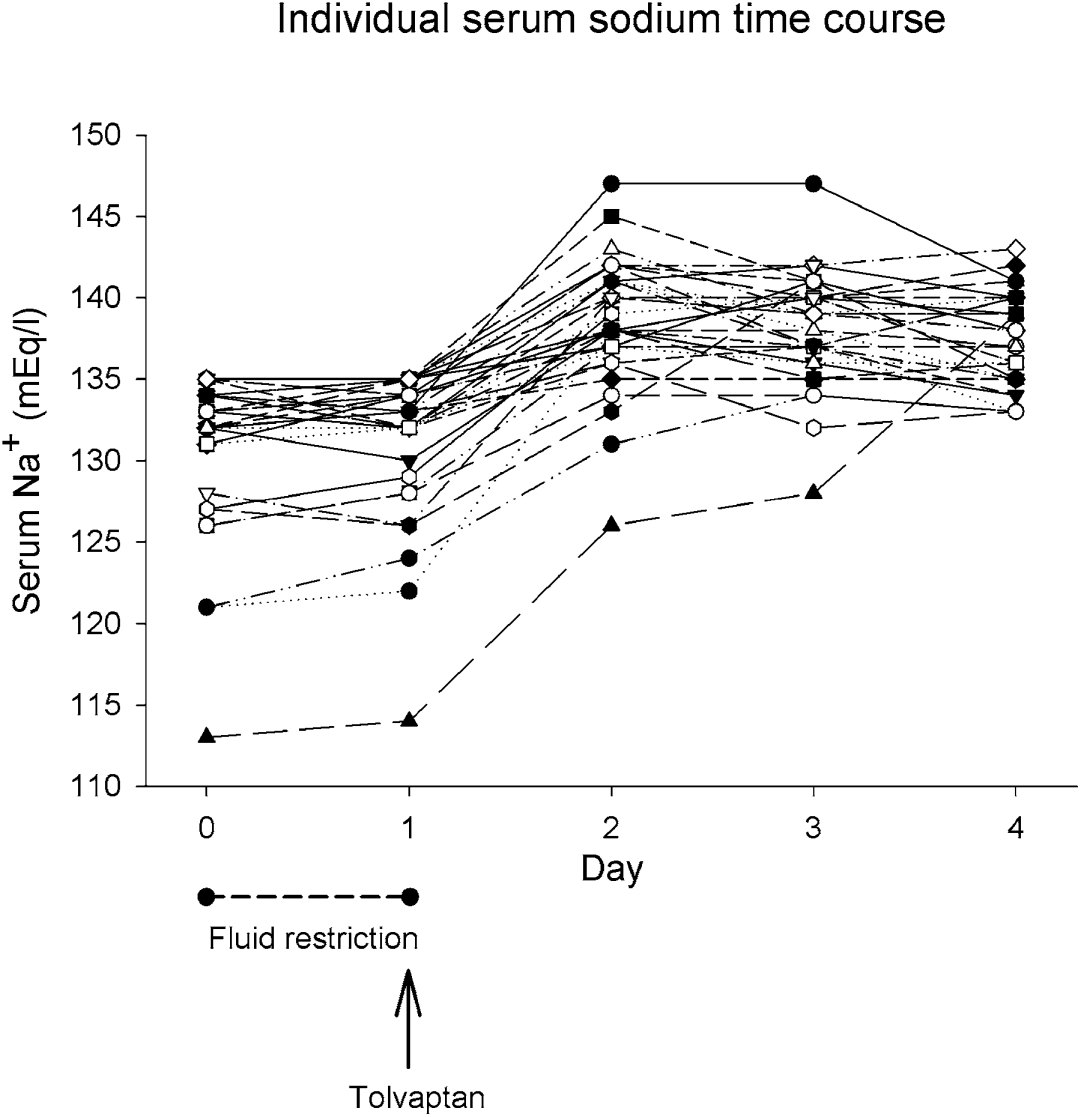
Individual patient time course of serum sodium data before and up to 72 h after Tolvaptan

**Fig. 2:**
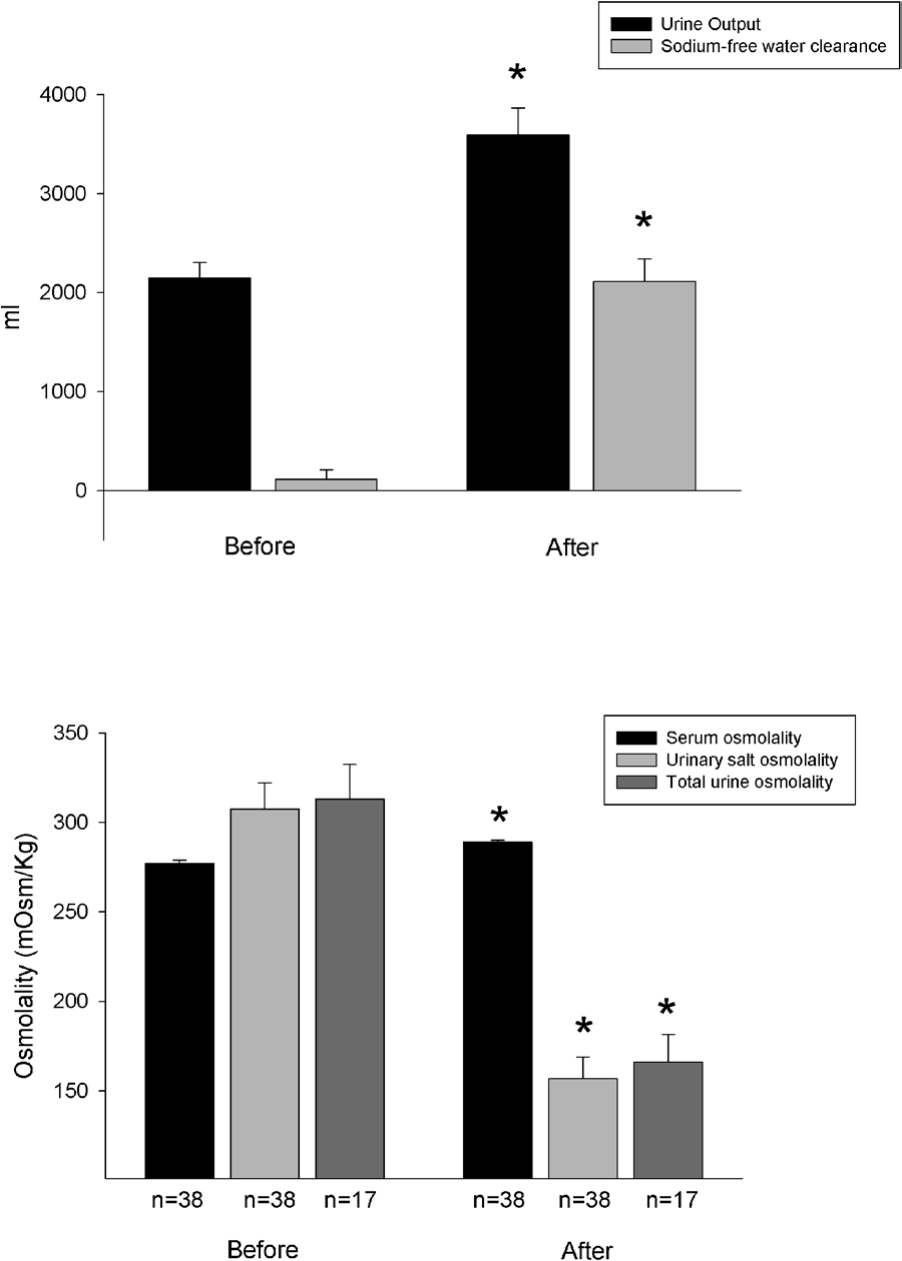
Changes in urine output and sodium-free water clearance (*upper panel*) and in serum and urine osmolality (*lower panel*) before and 24 h after Tolvaptan. Urine osmolality was calculated either as salt osmolality (i.e., the part of osmolality exerted by strong ions) and, for the subset of patients in whom urinary nitrogen determinations were available, as total urinary osmolality. *p < 0.01 before vs. after Tolvaptan

Before fluid restriction, mean baseline [Na^+^] was 133 [131; 134] (range 113–135) mmol/L. After a 24 h trial of fluid restriction, it was 133 [132; 135] (range 114–135) mmol/L (*p* = 0.431), and increased to 138 [137; 141] (range 128–147) mmol/L at 24 h after administration of Tolvaptan (*p* < 0.001). Increase in [Na^+^] was sustained at 48 and 72 h after administration of Tolvaptan (139 [137; 140] (range 126–147) and 137 [135; 140] (range 133–143) mmol/L, respectively; *p* < 0.001). Fig 1 shows individual patient time course of serum sodium data. Table 3 shows the comparison of serum and urine electrolytes, sodium and fluid balance, hemodynamics, and liver and kidney function tests before and 24 h after administration of Tolvaptan. Fig 2 shows the changes in urine output and sodium-free water clearance observed before and 24 h after administration of Tolvaptan, as well as the change in serum and urine osmolality. No subsequent doses of Tolvaptan were administered. [Na^+^] at ICU discharge was 139 [136; 140].

**Table 3:** Changes in electrolytes, hemodynamic, and biochemistry parameters before and after administration of Tolvaptan

	Before	After	*P*
Serum sodium concentration (mmol/l)	133 [132; 135]	138 [137; 141]	<0.001
Urine sodium concentration (mmol/l)	123.2 ± 36.5	54.9 ± 5.4	<0.001
Serum potassium concentration (mmol/l)	4.1 ± 0.4	4.2 ± 0.3	0.823
Urine potassium concentration (mmol/l)	32.2 ± 16.8	24.8 ± 15.1	0.015
Urine output (ml/24 h)	2149 ± 966	3593 ± 1673	<0.001
Sodium input (mmol/24 h)	159 [111; 214]	148 [115; 169]	0.824
Sodium balance (mmol/24 h)	−69 [143; 20]	−49 [−146; 37]	0.789
Sodium output (mmol/24 h)	236.5 [120; 311]	190.5 [119.5; 298.5]	0.654
Sodium-free water clearance (%)	9.6 [−12.0; 24.4]	59.9 [43.3; 76.6]	<0.001
Fluid balance (ml/24 h)	−200 [−950; 0]	−1300 [−2200; −550]	<0.001
Heart rate (1/min)	90.3 ± 18.2	92.1 ± 20.4	0.467
Mean arterial pressure (mmHg)	84.3 ± 13.9	82.7 ± 11.0	0.413
Central venous pressure (mmHg)	7.1 ± 3.6	6.1 ± 4.1	0.089
Patients on vasopressors	1/38	1/38	>0.999
Central venous oxygen saturation (%)	71.6 ± 7.4	70.9 ± 7.2	0.445
Albumin concentration (g/100 ml)	2.3 ± 0.3	2.6 ± 0.3	0.015
Hemoglobin concentration (g/100 ml)	10.6 ± 2.0	10.8 ± 2.0	0.098
Aspartate aminotransferase (IU/l)	59 ± 27	49 ± 22	0.114
Alanine aminotransferase (IU/l)	84 ± 55	80 ± 56	0.439
Bilirubin concentration (mg/100 ml)	1.8 ± 1.3	1.9 ± 1	0.986
Serum creatinine concentration (mg/100 ml)	0.9 ± 0.6	0.8 ± 0.5	0.053
Blood urea nitrogen (mg/100 ml)	23.6 ± 13.3	20.1 ± 11.5	0.015
Serum lactate concentration (mmol/l)	1.2 ± 0.7	1.0 ± 0.4	0.261
Serum glucose concentration (mg/100 ml)	126 ± 28	120 ± 21	0.194

Four patients (10.5 %) experienced rapid correction of hyponatraemia at 24 h; all patients increased their serum sodium of 14 mEq/L. All events were asymptomatic. No cases of hypokalemia or hyperkalemia occurred; 1 patient (2.6 %) developed mild hypernatremia ([Na^+^] 147 mEq/L). No other adverse events were documented.

### Determinants of serum sodium increase

Serum sodium (*R* = −0.622, *p* < 0.001), urine sodium (*R* = −0.345, *p* < 0.001), central venous blood oxygen saturation (*R* = 0.401, *p* = 0.013), and BUN (*R* = −0.416, *p* = 0.031) before Tolvaptan administration were all significantly correlated with the absolute increase in [Na^+^] 24 h after Tolvaptan administration.

## Discussion

Maintenance of plasma osmolality and sodium concentrations within tight limits is one of the most highly regulated parameters of body physiology [35]; osmotically induced variations in cell volume can exert adverse effects on multiple cellular functions [36]. In virtually every disease state, the presence of hyponatremia was found to be an independent risk factor for increased mortality [11]. Indeed, a recent study in a large number of hospitalized hyponatremic patients proposed revising the definition of hyponatremia to [Na^+^] < 138 mmol/L, because this was the level at which the association with increased mortality reached statistical significance [37].

Treating hyponatremia thus seems an important step in the management of patients, especially for the most critically ill. However, this entails choosing among several suboptimal therapies, i.e., fluid restriction, salt tablets, slow infusions of 3 % saline, furosemide, and urea [6]. In cases of hyponatremia with preserved sodium pool, fluid restriction represents the cheapest, first-line treatment, despite the almost complete lack of a supportive evidence base [11]. The other associated therapeutic options, however, are represented by drugs with limited efficacy or a doubtful physiologic rationale, in that they mainly act on body sodium pool, while it is the water pool that is primarily altered. The recent development of the new pharmacologic approach of aquaresis— i.e., electrolyte-free water excretion— allows for removal of water not accompanied by elimination of sodium and other electrolytes [38].

Tolvaptan is an oral, competitive, selective vasopressin V2-receptor antagonist. It was shown to be effective in reversing hyponatremia with preserved total body sodium pool ([Na^+^] < 135 mmol/L) in the SALT-1 and SALT-2 trials [24]. Pooled results for all patients showed changes in average daily serum sodium of 4.0 mmol/L vs 0.4 mmol/L for placebo at day 4. A post hoc analysis of the phase III EVEREST trial demonstrated that patients hospitalized for heart failure with baseline hyponatremia who received Tolvaptan had an adjusted mean length of stay shorter compared with that for patients receiving placebo [25]. Tolvaptan was also shown to reduce hospital length of stay vs. placebo among patients with SIAD in the SALT-1 and -2 trials [39]. A recent meta-analysis on 11 trials (1094 patients) showed how the use of vaptans resulted in a 24-h net increase in [Na^+^] of 3.3 mmol/L relative to the control group [17].

However, the published experience in current clinical practice and in patients not enrolled in randomized trials remains limited, particularly in the field of critical care medicine. Furthermore, clinical factors which can predict the extent of the response are not completely defined. The present investigation is one of the very few reporting experience of vaptans in critically ill patients, previous studies being case reports, or open label randomized studies assessing interest of Conivaptan in neuro-ICU patients with [40] or without [41] hyponatremia.

In this case series, we present a mixed cohort of critically ill patients who were either admitted to the ICU or developed during their stay a moderate hypotonic hyponatremia. The patients had no history of reduction in their total sodium pool, nor did they present any sign or symptom of overt hypovolemia (as evaluated by their values of hemodynamic parameters, urine output, blood lactate, ScvO2, skin perfusion); urine sodium was above the normal range. However, the average concentration of 10 g/100 ml hemoglobin associated with 23 g/l albumin (Table 2), widely tolerated combination of hypoalbuminemia/anemia frequently seen in critically ill patients [42], might have contributed to the activation of AVP (given the altered circulating blood volume), besides the well-known factors (acute respiratory failure, pneumonia, trauma, mechanical ventilation, history of COPD) [8] that are nonetheless present in the case-mix.

After a trial of fluid restriction, Tolvaptan was administered enterally (via nasogastric tube) and, given the lack of previous experience with this drug in critically ill patients, we decided to use lower doses than generally used (on average, 7.5 mg). Even so, we report a rapid and effective response (average increase of 7 mmol/l in 24 h) that nonetheless lasted up to 72 h after the first administration, again underscoring the feasibility of the enteral route for critically ill patients [43, 44]. Tolvaptan significantly increased [Na^+^] and reduced urine sodium, with no changes in either plasma or urine potassium. Moreover, sodium input and sodium balance were unchanged from before and after administration of the drug; still, urine output increased about 1.5 l, indicating free water diuresis (Fig. 2). The increased diuresis and the consequent negative fluid balance were not associated with any hemodynamic modification, nor did they induce hypoperfusion, again indicating the previously increased total body water pool and a likely osmotically driven reduction in the increased cell water content after administration of Tolvaptan. The decrease in urine [Na^+^] concentration was likely a dilutional effect of the increased urine water content, as total urine sodium output did not change significantly.

Only about 10 % of patients experienced a daily increase in plasma sodium >12 mmol/l. Since overly rapid correction of hyponatremia risks iatrogenic brain damage, a 1-day increase of 12 mmol/L/d is generally considered a safe threshold [11]. However, the risk of central pontine myelinolysis varies depending on several factors, and it is unlikely to occur if serum Na concentration is >120 mEq/L [11]. One patient became mildly hypernatremic (147 mEq/L), and despite the fact that he developed no neurological symptoms, this should be acknowledged as development of hypernatremia is a risk factor for osmotic demyelination [11]. Indeed, none of these patients reported abnormalities to the neurologic examination, and particularly no cases of central pontine myelinolysis or any other severe neurological damage were seen. Moreover, this rate of correction is in line with other data on hyponatremia with preserved sodium pool in larger group of patients studied in the Hyponatremia Registry [5], or in patients treated with Tolvaptan [17], and lower than reported with the use of intravenous Conivaptan [33], thus suggesting a low prevalence of such side-effect with the low doses we used or a less drastic effect of enteral administration of the drug. We did not report any other relevant side-effect, and specifically, we did not see any short-term alteration in liver or kidney function tests.

We observed a certain degree of variability in the response to Tolvaptan administration. To better elucidate the potential mechanisms, and to possibly provide clinicians with predictors of the response to Tolvaptan, we searched for variables associated to the degree of the effect. As already reported [23, 33, 45], baseline [Na^+^] was negatively associated with the subsequent rise in [Na^+^] resulting from treatment, i.e., the lower the baseline [Na^+^], the bigger the response. This could merely reflect the fact that subjects with higher baseline [Na^+^] have less room for improvement, and likely reflect a lower degree of AVP activation. Furthermore, our study shows how baseline urine sodium has a negative association with [Na^+^] rise. This likely depends from a higher AVP activation leading to an elevated urine sodium, that might have necessitated higher doses of Tolvaptan for the same effect to take place, and is in line with the competitive nature of the AVP antagonism exerted by the drug. The positive correlation with ScvO2 might mean that patients with a relatively more increased body water pool tend to have a bigger response. Eventually, the negative correlation with BUN, as already seen with intravenous Conivaptan [33], likely depends on the fact that hypouraemia is a known feature of SIAD [46] (decreased proximal tubular reabsorption of urea and increased GFR resulting from expansion of body water pool [47]). One could speculate that subjects with the lowest BUN levels carry the largest increase in whole body water content, greatest extracellular fluid expansion, highest GFR, lowest proximal tubular reabsorption, and greatest distal delivery of substrate, thereby making them more responsive to water diuresis.

### Study limitations

Potential limitations of the present study include the small sample population in a heterogeneous population of critically ill patients and the lack of statistical power in detecting significant side effects or complications of unwarranted fast corrections of hyponatremia. However, we succeeded in testing our primary hypothesis, which showed a significant improvement in [Na^+^] without short-term negative effects in a real-life scenario. Secondly, as a single-center study with no control group for comparison, the generalizability of the present results is limited. Moreover, given the observational nature of the study, the statistical associations between serum sodium concentration and outcome cannot be considered as causal relationship. Furthermore, factors correlated with sodium increase following Tolvaptan administration were evaluated using bivariate correlation; however, the reported factors might be either predictors of confounding factors not taken into account because of the limited sample size, which precluded any such correction. Larger prospective, placebo-controlled, randomized studies are needed to confirm the effects of Tolvaptan treatment as well as its cost-effectiveness. Finally, the long-term effects of low-dose Tolvaptan in this population were not tested in the present study.

## Conclusions

In conclusion, this is the first case series to suggest that enteral administration of oral Tolvaptan is an effective aquaretic for the treatment of hyponatremia with preserved total body sodium pool in a heterogeneous cohort of critically ill patients. Further prospective well-designed studies are required to determine if the administration of Tolvaptan to critically ill hyponatremic patients can improve their clinical outcome.

## Additional file

10.1186/s13613-015-0096-2 **Table S1.** Baseline characteristics and primary and secondary outcomes in patients receiving either 7.5 or 15 mg of Tolvaptan. **Table S2.** Changes in electrolytes, hemodynamic and biochemistry parameters before and after administration of Tolvaptan in patients receiving either 7.5 or 15 mg.

## Abbreviations

ICU: intensive care unit
BUN: blood urea nitrogen
AVP: arginine vasopressin
SIAD: syndrome of inappropriate antidiuresis
GFR: glomerular filtration rate
[Na^+^]: serum sodium concentration
