# Supplementary material for: Tolvaptan for hyponatremia with preserved sodium pool in critically ill patients
Source: Ann Intensive Care. 2016 Jan 4;6:1. doi: 10.1186/s13613-015-0096-2 (PMC4700037; doi:10.1186/s13613-015-0096-2)
Supplement: Supplementary file 1 — 10.1186/s13613-015-0096-2 Table S1. Baseline characteristics and primary and secondary outcomes in patients receiving either 7.5 or 15 mg of Tolvaptan. Table S2. Changes in electrolytes, hemodynamic and biochemistry parameters before and after administration of Tolvaptan in patients receiving either 7.5 or 15 mg. [file 13613_2015_96_MOESM1_ESM.doc]

**Tolvaptan for hyponatremia with preserved sodium pool in critically ill patients**

**ELECTRONIC SUPPLEMENTARY MATERIAL**

Michele Umbrello^a,b^ (email: michele.umbrello@fastwebnet.it)

Elena S. Mantovani^a^ (email: elenasilvia.mantovani@ao-sanpaolo.it)

Paolo Formenti^a^ (email: formenti.paolo@fastwebnet.it)

Claudia Casiraghi^b^ (email: claudia.casiraghi@unimi.it)

Davide Ottolina^b^ (email: davide.ottolina@unimi.it)

Martina Taverna^b^ (email: martina.taverna@unimi.it)

Angelo Pezzi^a^ (email: angelo.pezzi@ao-sanpaolo.it)

Giovanni Mistraletti^a,b^ (email: giovanni.mistraletti@unimi.it)

Gaetano Iapichino^a,b^ (email: g.iapichino@unimi.it)

From the: ^a^Unità Operativa di Anestesia e Rianimazione, Azienda Ospedaliera San Paolo - Polo Universitario, Milano, Italy; ^b^Dipartimento di Fisiopatologia Medico-Chirurgica e dei Trapianti, Università degli Studi di Milano, Milano, Italy

**Corresponding author and address for reprints:**

Michele Umbrello, MD

UO Anestesia e Rianimazione

A.O. San Paolo - Polo Universitario

Via A. Di Rudinì, 8 - 20142 Milano - Italy

Email michele.umbrello@ao-sanpaolo.it

SUPPLEMENTARY TABLES

Table S1 – Baseline characteristics and primary and secondary outcomes in patients receiving either 7.5 or 15 mg of Tolvaptan

|  | **7.5 mg** | **15 mg** | **p** |
| --- | --- | --- | --- |
| Weight (kg) | 70.4±14.4 | 86.8±28.6 | 0.023 |
| Ideal body weight (Kg) | 62.3±5.9 | 65.9±7.9 | 0.118 |
| Na prima samsca (mEq/L) | 133 [131.5; 134] | 133 [131; 135] | 0.656 |
| Na admission (mEq/L) | 135 [131; 138] | 135 [131; 141] | 0.705 |
| Age (years) | 53±14 | 54±17 | 0.824 |
| SAPS II | 24.7±13.0 | 27.5±10.5 | 0.486 |
| SOFA | 3.4±2.8 | 4.9±3.9 | 0.275 |
|  |  |  |  |
| Primary outcome |  |  |  |
| ≥4 mmol/l increase in serum sodium over baseline at 24h – n (%) | 18 (75%) | 13 (93%) | 0.177 |
|  |  |  |  |
| Secondary outcomes |  |  |  |
| Absolute increase in serum sodium over baseline at 24h – mmol/l | 6.8±3.9 | 6.5±2.4 | 0.772 |
| Absolute increase in serum sodium over baseline at 72h – mmol/l | 5.4±4.1 | 5.6±3.8 | 0.835 |
| Absolute reduction in urine sodium over baseline at 24h – mmol/l | -72.3±41.5 | -61.5±37.4 | 0.431 |
| ≥12 mmol/l increase in serum sodium over baseline at 24h – n (%) | 4 (16.7%) | 0 (0%) | 0.106 |
| Average hourly increase in serum sodium – mmol/l*h | 0.28±0.16 | 0.27±0.1 | 0.772 |

Table S2 – Changes in electrolytes, hemodynamic and biochemistry parameters before and after administration of Tolvaptan in patients receiving either 7.5 or 15 mg

|  | **7.5 mg** | | | **15 mg** | | |
| --- | --- | --- | --- | --- | --- | --- |
|  | **Before** | **After** | **p** | **Before** | **After** | **p** |
| Serum sodium concentration (mmol/l) | 133 [132; 134] | 138 [136.5; 141] | <0.001 | 134 [130; 135] | 138 [137; 141] | <0.001 |
| Urine sodium concentration (mmol/l) | 131.5±38.7 | 59.3±34.7 | <0.001 | 109.0±28.3 | 47.5±29.5 | <0.001 |
| Serum potassium concentration (mmol/l) | 4.2±0.3 | 4.2±0.2 | 0.579 | 4.0±0.4 | 4.2±0.3 | 0.432 |
| Urine potassium concentration (mmol/l) | 30.9±17.6 | 23.6±16.8 | 0.100 | 34.3±15.9 | 26.7±12.3 | 0.048 |
| Urine output (ml/24h) | 2155±838 | 3485±1756 | <0.001 | 2138±1187 | 3775±1565 | <0.001 |
| Sodium input (mmol/24h) | 163 [88; 228] | 150 [119; 246] | 0.476 | 155 [126; 187] | 144 [115; 163] | 0.249 |
| Sodium balance (mmol/24h) | -76 [-157; 5] | -80 [-148; -10] | 0.614 | -40 [-130; 92] | 2 [-130; 45] | 0.807 |
| Sodium output (mmol/24h) | 217 [134.5; 306] | 143.5 [145; 270] | 0.961 | 236.5 [61; 311] | 158.5 [38; 318] | 0.470 |
| Sodium-free water clearance (%) | -0.4 [-15.8; 15.5] | 60.0 [35.8; 71.1] | <0.001 | 21.7 [10.4; 33.6] | 64.3 [47.1; 83.5] | <0.001 |
| Fluid balance (ml/24h) | -200 [-375; 0] | -625 [-1750; -400] | <0.001 | -750 [-1730; 0] | -2200 [-2850; -1150] | <0.001 |
| Heart rate (1/min) | 91.9±18.0 | 95.8±23.0 | 0.246 | 87.5±18.9 | 85.7±13.2 | 0.618 |
| Mean arterial pressure (mmHg) | 87.1±12.1 | 84.0±10.2 | 0.090 | 79.4±16.0 | 80.4±12.5 | 0.832 |
| Central venous pressure (mmHg) | 6.1±3.6 | 5.4±3.9 | 0.193 | 8.4±3.3 | 7.1±4.4 | 0.285 |
| Central venous oxygen saturation (%) | 72.9±6.8 | 71.2±7.0 | 0.215 | 69.4±8.1 | 70.3±7.9 | 0.464 |
| Albumin concentration (g/100 ml) | 2.5±0.2 | 2.7±0.4 | 0.148 | 2.2±0.4 | 2.4±0.4 | 0.072 |
| Hemoglobin concentration (g/100 ml) | 10.9±2.1 | 11.1±2.1 | 0.178 | 9.9±1.7 | 10.2±1.7 | 0.345 |
| Aspartate aminotranspherase (IU/l) | 42.2±21.1 | 35.6±12.2 | 0.256 | 58.2±26.7 | 52.5±26.5 | 0.239 |
| Alanine aminotranspherase (IU/l) | 64.7±43.8 | 49.6±24.9 | 0.718 | 76.9±58.8 | 90.7±65.8 | 0.536 |
| Bilirubin concentration (mg/100 ml) | 1.6±1.4 | 1.2±0.5 | 0.661 | 2.1±1.3 | 1.8±1.2 | 0.754 |
| Serum creatinine concentration (mg/100 ml) | 0.6±0.2 | 0.6±0.2 | 0.443 | 1.0±0.7 | 0.9±0.7 | 0.082 |
| Blood urea nitrogen (mg/100 ml) | 17.0±5.0 | 15.8±4.9 | 0.243 | 25.6±15.1 | 22.2±13.7 | 0.036 |
| Serum lactate concentration (mmol/l) | 1.2±0.8 | 1.1±0.5 | 0.623 | 1.2±0.8 | 0.9±0.3 | 0.254 |
| Serum glucose concentration (mg/100 ml) | 121.6±25.9 | 119.1±20.2 | 0.663 | 133.4±30.4 | 121.3±23.9 | 0.128 |
